# Evaluation of Microbiological Susceptibility and Long-term Adhesive Properties to Dentin of Primers with *Terminalia catappa* Linn

**DOI:** 10.3290/j.jad.b5199073

**Published:** 2024-04-11

**Authors:** Sylvia Rejanne Carvalho Lobão, Rammon de Faria Nonato, Pedro Henrique de Aguiar Moreira, Aline Michelle Silva Mendonça, Milena Trovão, Michel Wendlinger Cantanhede Ferreira, Barbara Emanoele Costa Oliveira, Luis Claudio Nascimento da Silva, Letícia Machado Gonçalves, Andres Felipe Millan Cardenas, Viviane Hass, Alessandro D. Loguercio, Fabiana Suelen Figuerêdo de Siqueira

**Affiliations:** a Dentist, Postgraduate Program in Dentistry, CEUMA University, São Luis, Maranhão, Brazil. Designed testing assembly, performed bond strength experiments.; b PhD Student, Postgraduate Program in Dentistry, CEUMA University, São Luis, Maranhão, Brazil. Designed testing assembly, performed bond strength and microbiological experiments.; c MS Student, Postgraduate Program in Dentistry, CEUMA University, São Luis, Maranhão, Brazil. Performed bond strength and nanoleakage experiments.; d Dentist, Postgraduate Program in Microbial Biology, CEUMA University, São Luis, Maranhão, Brazil. Performed microbiological experiments.; e PhD Student of Postgraduate Program in Dentistry, CEUMA University, São Luis, Maranhão, Brazil. Co-wrote paper and proofread the manuscript.; f PhD Student of Department of Restorative Dentistry, Ponta Grossa State University, Ponta Grossa, Paraná, Brazil. Performed in situ degree of conversion and nanoleakage experiments.; g Professor, Postgraduate Program in Dentistry, CEUMA University, São Luis, Maranhão, Brazil. Performed microbiological experiments and co-wrote paper.; h Professor, Postgraduate Program in Microbial Biology, CEUMA University, São Luis, Maranhão, Brazil. Designed testing assembly, performed microbiological experiments, and proofread the manuscript.; i Professor, Department of Dentistry, University Federal of Maranhão, São Luis, Maranhão, Brazil. Research idea, designed testing assembly, performed microbiological experiments, and proofread the manuscript.; j Professor, Postgraduate Program in Dentistry, CEUMA University, São Luis, Maranhão, Brazil. Research idea, designed testing assembly, co-wrote paper, contributed substantially to the discussion and proofread the manuscript.; k Professor, School of Dentistry, University of Missouri, Kansas City, MO, USA. Contributed substantially to the discussion and proofread the manuscript.; l Professor, Department of Restorative Dentistry, Ponta Grossa State University, Ponta Grossa, Paraná, Brazil. Provided consulting for statistical analysis and contributed substantially to the discussion and proofread the manuscript.; m Professor, Postgraduate Program in Dentistry, CEUMA University, São Luis, Maranhão, Brazil. Research idea, designed testing assembly, co-wrote paper, contributed substantially to the discussion and proofread the manuscript.

**Keywords:** microbial degradation, dentin adhesives, scanning electron microscopy, flavonoids

## Abstract

**Purpose:**

To investigate the antibacterial effects of *Terminalia catappa* Linn (TCL) leaf extracts at different concentrations and the effects of these extracts used as primers on the long-term adhesive properties of two universal adhesives.

**Materials and Methods:**

After extract preparation, the antimicrobial and antibacterial activities of TCL against *Streptococcus mutans* (UA 159) were assessed in microdilution assays to provide the minimal inhibitory concentration (MIC) and minimal bactericidal concentration (MBC). Additionally, to provide quantitative data on the ability of TCL extract to reduce cell viability, colony forming units (CFU) were counted. To examine adhesive properties, 288 human molars were randomly assigned to 32 experimental conditions (n = 9) according to the following variables: (1) treatment agent: negative control (untreated surface), and primers at concentrations of 1xMIC, 5xMIC, and 10xMIC; (2) adhesives: Scotchbond Universal (SBU) and Futurabond Universal (FBU); (3) adhesive strategy: etch-and-rinse (ER) or self-etch (SE); and (4) storage time: 24 h or after 2 years. Primers were applied for 60 s, upon which the teeth were incrementally restored and sectioned into adhesive-dentin bonded sticks. These were tested for microtensile bond strength (μTBS) and nanoleakage (NL) after 24-h and 2-year water storage, as well as in-situ degree of conversion (DC) at 24 h. The chemical profile of the hybrid layer was determined via micro-Raman spectroscopy. Biofilm assay data were analyzed using the Kruskal-Wallis test; the pH of culture media and the chemical profile were analyzed by one-way ANOVA. The adhesive properties (µTBS, NL, DC) were evaluated using a four-way ANOVA and Tukey’s test. Significance was set at 5%.

**Results:**

Similar values of MIC and MBC were observed (2 mg/ml), showing bactericidal potential. CFU analysis demonstrated that concentrations of 5xMIC and 10xMIC significantly inhibited biofilm formation (p < 0.001). The application of the TCL primer at all concentrations significantly increased the immediate μTBS and DC, and decreased the immediate NL values when compared to the control group (p < 0.05), regardless of the adhesive and adhesive strategies. Despite an increase in the NL values for all groups after 2 years (p > 0.05), in groups where the TCL primer was applied, the μTBS remained constant after 2 years for both adhesives, while a decrease in the μTBS was observed in the control groups (p < 0.05). Usually, 10xMIC showed better results than 1xMIC and 5xMIC (p < 0.05). The application of TCL promoted cross-linking; cross-linking rates increased proportionally to the concentration of TCL (p < 0.05).

**Conclusion:**

Primers containing TCL promoted bactericidal and bacteriostatic action, as well as cross-linking with dentin, while maintaining the adhesive properties of the adhesive-dentin interface after 2 years of water storage.

Resin composite restorations are currently the most widespread option for restorative treatments performed on posterior teeth.^43,53^ However, secondary caries is considered one of the most frequent causes of failure. For instance, Askar et al^6^ showed a higher risk of secondary caries when simplified adhesives were used, and Nedeljkovic et al^45^ found that 66% of resin composite restorations required replacement due to the presence of secondary caries.

In caries progression, the dentin organic matrix demineralizes, making it prone to slow hydrolytic degradation by host collagenolytic enzymes, matrix metalloproteinases (MMPs), and cysteine cathepsins.^62^ This collagenolytic activity in caries-affected dentin may contribute to adhesive interface failure, resulting in greater expenditures for dental services.^62,33^

To prevent the formation of secondary caries and avoid degradation of the tooth-composite interface, many different substances have been examined. For instance, different bioactive compounds, such as calcium phosphate, silver diamine fluoride, and bioactive glass,^9,12,63^ non-natural substances with antimicrobial and anti-MMP action, such as chlorhexidine, tetracyclines, and 12-methacryloxydodecylpyridinium bromide (MDPB), as well as natural substances, e.g., proanthocyanidins, have been evaluated with excellent in-vitro results.^7,22,49,71^ However, their clinical performance remains questionable.^4,20,34,49^

For example, using proanthocyanidins associated with adhesives causes a reduction in the mechanical properties of the adhesive^25^ and stains dentin with a typical dark brownish color.^7,44^ Both these factors lead to a higher number of failures in composite restorations performed with adhesives associated with proanthocyanidins.^22,34^ Considering that most agents are of synthetic origin, plant extracts have provided a new alternative for the development of therapeutic substances with antimicrobial and anti-enzymatic potential to reduce restorative treatment costs and promote the long-term stability of adhesive restorations.^19,44^

Plant extracts from *Terminalia catappa* Linn (TCL) have attracted attention for their antiviral, anti-inflammatory, and anti-enzymatic activities^3,68^ owing to their phytoconstituents, such as flavonoids, carotenoids, and phenolic compounds.^3^ In dentistry, TCL extracts have been shown to have a positive effect in decreasing bacterial colonization of dental prostheses.^24^ However, according to the authors’ knowledge, no studies have evaluated the antibacterial effect of TCL extract against *Streptococcus mutans* or its effect on the bonding properties of adhesives to dentin.

Thus, the first aim of the present study was to determine the susceptibility of *Streptococcus mutans* to the TCL extract, expressed as minimal inhibitory concentrations (MIC) and minimal bactericidal concentrations (MBC). The second aim was to evaluate the effect on adhesive-dentin interfacial properties (microtensile bond strength [μTBS], nanoleakage [NL], and in-situ degree of conversion [DC]) of different multiples of MIC of the TCL extract applied as pre-treatment (primers) with two universal adhesives. The chemical profile of the hybrid layer was also evaluated. The hypotheses tested were as follows: 1) *Streptococcus mutans* would be susceptible to TCL extract; 2) the TCL extract primer would affect the adhesive properties to dentin immediately (24 h) or after 2 years of water storage; 3) the TCL extract primer would have some cross-linking effect in dentin after 24 h.

## Materials and Methods

### Collection, Botanical Identification, and Extract Preparation

*T. catappa* was cultivated in an experimental field at CEUMAuniversity (São Luis, MA, Brazil). Plant material was collected from September 2019 to November 2019; only the leaves were used. The exsiccate was prepared and sent to a special laboratory for botanical identification under voucher template no. 01062. The leaves were dried in an oven (Dabi Atlante; Ribeirão Preto, SP, Brazil) with air circulation at 37°C for one week, followed by milling (Marconi Equipamentos; Piracicaba, SP, Brazil). Approximately 200 g of dry and ground material were macerated with approximately 800 ml of 70% ethanol (Onixlimp; Santo André, SP, Brazil) for 24 h at 37°C. This process was repeated four times, and the obtained extract was filtered and concentrated using a rotary evaporator (Lutech; São José do Rio Preto, SP, Brazil).

The crude hydroalcoholic extract (purity 98%) was lyophilized and then resuspended in 600 ml of MeOH/H_2_O (80:20, v/v) (Rauter Química; Gravataí, RS, Brazil), followed by liquid-liquid fractionation with hexane (Merck; Darmstadt, Germany), ethyl acetate (Merck), and n-butanol (Merck), resulting in three fractions with different polarities: hexane fraction, which was the least polar; ethyl acetate with intermediate polarity; and the most polar n-butanol fraction.^59^ As previously observed, the n-butanol fraction presented the best antibiofilm activity.^24,46,61^ The n-butanol fraction was concentrated using a rotary evaporator (Büchi Labortechnik; Flawil, Switzerland) and then lyophilized, resulting in a fine powder (VirTis Lyophilizer Tray Dryer, ATS; Warminster, PA, USA). This powder was weighed and stored in an amber-colored bottle until further testing. One hour before use, 100 mg of TCL powder was dissolved in 1000 µl of sterile distilled water.

### Antibacterial Properties

#### Microbiological susceptibility tests

Minimal inhibitory concentration (MIC) and minimal bactericidal concentration (MBC) analyses were performed to assess the susceptibility of planktonic *S. mutans* cells to the TCL extract. As a positive control group, 0.12% chlorhexidine was used, while the reference strain *S. mutans* UA 159 was used to reactivate the microorganism and prepare the inoculum. It is worth mentioning that CHX was only used in the evaluation of antibacterial properties. This strain was reactivated in its original culture in a BHI (brain heart infusion) plate at 37°C for 24 h under microaerophilic conditions. To prepare the inoculum, colonies were transferred to a tube containing 1000 µl of sterile saline solution (0.85% NaCl) and the turbidity of the solution was adjusted using the 0.5 McFarland standard, ensuring a suspension of 1.108 ≅ 5105 cells/ml.

MIC was determined by the microdilution method in broth using a 96-well plate, as recommended by the Clinical and Laboratory Standards Institute (CLSI).^52^ The initial 100 ml/mg concentration of the TCL extract was dissolved in 920 µl of sterile distilled water, following the CLSI protocol.^52^ Thus, 100 µl of the TCL extract and 100 µl of the medium (standardized inoculum and BHI) were added to the 96-well plate to perform serial dilutions. The wells containing the different dilutions of TCL extract, controls (positive and negative), and the inoculum were incubated under anaerobic conditions at 37°C for 24 h. The test readout was performed by visual comparison, and the MIC corresponded to the lowest concentration that prevented the visible growth of planktonic cells. Each concentration in the previous test that presented no visible growth was inoculated into a BHI plate.

MBC was determined as the lowest concentration of the antimicrobial agent which killed 99.9% of the initial bacterial population. After MIC determination (termed 1xMIC), 10-μl aliquots of bacterial suspensions from the wells with concentrations of 1xMIC, 5xMIC, and 10xMIC were seeded on BHI agar plates and incubated under anaerobic conditions for 48 h at 37°C with 5% CO_2_. Three independent experiments were performed for MBC and MIC.

#### Antibiofilm activity of T. catappa L. to Streptococcus mutans

Considering that the TCL extract proposed in this study is intended for the treatment of carious lesions, *S. mutans* was chosen for the antimicrobial tests. Thus, to evaluate the antibiofilm activity of TCL, 200 μl of BHI culture medium, *S. mutans* bacterial inoculum at a concentration of 1x10^6^ CFU/ml, glucose at a final concentration of 1%, and TCL extract at concentrations of 1xMIC, 5xMIC, and 10xMIC were added in triplicate to 12-well microplates. Additionally, a group (*S. mutans* + BHI + 1% glucose) without TCL extract was used as a positive control.^51^ Subsequently, circular coverslips (13 mm diameter) were inserted into the wells using sterile forceps. After being incubated for 24 h at 37°C in 10% CO_2_, the circular coverslips were carefully removed, washed twice with saline solution, and fixed with 200 μl of methanol for staining with 0.1% crystal violet. Following staining, the coverslips were evaluated using a Zeiss Flow Microscope (Zeiss; Oberkochen, Germany).^ 51^

Additionally, to provide quantitative data on the ability of TCL extract to reduce cell viability, *S. mutans* biofilms (n=9/group) were cultured in 96-well plates^23^ in BHI supplemented with 1% glucose for 24 h. Pre-determined concentrations of extract (1xMIC, 5xMIC, and 10xMIC) and no treatment (control) were tested. For this, the bacterial inoculum was adjusted (OD600 nm = 0.5), mixed with the antimicrobial treatments (1:1 v/v), and incubated for 24 h at 37ºC in an atmosphere of 10% CO_2_. Afterward, biofilms were collected, and cell viability was determined by serial dilution and CFU counting. Then, biofilms were incubated with resazurin (0.03%, 37ºC, 90 min) to qualitatively evaluate the metabolic activity of living cells,^16^ which is indicated by a color change from blue to red.^58^ Finally, a pool of 3 wells from each biological replicate was prepared for pH measurement using a pH meter calibrated with pH 4.0 and pH 7.0 buffers.^15^

### Evaluation of Adhesive-Dentin Interface

#### Tooth preparation and experimental design

Two hundred eighty-eight caries-free human molars were used after approval from the CEUMA university ethics committee (#3.813.682). The teeth obtained were disinfected with 0.5% chloramine and stored in distilled water to be used within 6 months after extraction. The occlusal third of the crown was removed from all teeth with a diamond saw (Isomet, Buehler; Lake Bluff, IL, USA) under water cooling, thereby exposing the occlusal dentin surface, which was then sanded with 600-grit silicon carbide paper (3M Oral Care; St Paul, MN, USA). Next, the 288 teeth were randomly allocated to 32 experimental conditions (n = 9) for the adhesive-dentin bonding tests according to the following variables: (1) treatment agent: control (distilled water), or aqueous solution with TCL extract of 1xMIC, 5xMIC, and 10xMIC; (2) adhesives: Scotchbond Universal (SBU; 3M Oral Care); Futurabond U (FBU, VOCO; Cuxhaven, Germany); (3) adhesive strategy: etch-and-rinse (ER) or self-etch (SE); and (4) storage time: 24 h and 2 years. The materials used, batch numbers, composition, and application modes are listed in Table 1.

**Table 1:** Adhesives, experimental groups, and application mode

Adhesive	Experimental groups	Adhesive application*	
		ER	SE
Scotchbond Universal adhesive (SBU, 3M Oral Care; St Paul, MN, USA)	Control	Apply etchant for 15 s Rinse for 10 s Air dry 5 s Keep slightly moist for the application of the adhesive Apply adhesive as for the SE mode	Apply the adhesive to the entire preparation with a microbrush and rub it in for 20 s. If necessary, re-wet the disposable applicator during treatment Direct a gentle stream of air over the liquid for about 5 s until it no longer moves and the solvent is evaporated completely Light cure for 10 s at 1200 mW/cm^2^
	1xMIC**	Etch for 15 s Rinse for 10 s Air dry for 5 s Keep slightly moist Apply an aqueous solution of *T. catappa* L. actively for 60 s Air dry for 5 s and keep the surface slightly moist Apply adhesive as for the SE mode	Keep slightly moist Apply an aqueous solution of *T. catappa* L. actively for 60 s Air dry for 5 s and keep the surface slightly moist Apply the adhesive to the entire preparation with a microbrush and rub it in for 20 s. If necessary, re-wet the disposable applicator during treatment Direct a gentle stream of air over the liquid for about 5 s until it no longer moves, and the solvent is evaporated completely Light cure for 10 s at 1200 mW/cm^2^
	5xMIC		
	10xMIC		
Futurabond U (FBU, VOCO; Cuxhaven, Germany)	Control	Apply etchant for 15 s Rinse for 10 s Air dry 5 s Keep slightly moist for the application of the adhesive Apply adhesive as for the SE mode	Apply the adhesive to the entire preparation with a microbrush and rub it for 20 s. If necessary, re-wet the disposable applicator during treatment Direct a gentle stream of air over the liquid for about 5 s until it no longer moves, and the solvent is evaporated completely Light cure for 10 s at 1200 mW/cm^2^
	1xMIC	Etch for 15 s Rinse for 10 s Air dry 5 s Keep slightly moist Apply an aqueous solution of *T. catappa* L. actively for 60 s Air dry for 5 s and keep the surface slightly moist Apply adhesive as for the SE mode	Keep slightly moist Apply an aqueous solution of *T. catappa* L. actively for 60 s Air dry for 5 s and keep the surface slightly moist Apply the adhesive to the entire preparation with a microbrush and rub it in for 20 s. If necessary, re-wet the disposable applicator during treatment Direct a gentle stream of air over the liquid for about 5 s until it no longer moves, and the solvent is evaporated completely Light cure for 10 s at 1200 mW/cm^2^
	5xMIC		
	10xMIC		

* Adhesives were applied according to the recommendations of their respective manufacturers; ** MIC: minimal inhibitory concentration.

#### Restorative procedure and specimen preparation

In the ER strategy, 37% phosphoric acid gel (Condac37, FGM; Joinville, SC, Brazil) was applied to the dentin surfaces for 15 s, rinsed with water for 30 s, and gently air dried for 5 s using an air syringe at a distance of 10.0 cm, with the dentin surface kept slightly moist. After etching, the dentin surfaces were treated with each experimental primer based on the MIC of the TCL extract, according to the experimental design. Distilled water was used for the control group. All aqueous solutions of TCL primers were vigorously applied for 60 s with a microbrush (KG Brush, KG Sorensen; Cotia, SP, Brazil) and kept slightly moist for application of the adhesive.

In the SE strategy, distilled water and an aqueous solution of each concentration of TCL primer were applied before using the universal adhesive. Subsequently, the adhesives were applied to all groups according to the respective manufacturer’s recommendations (Table 1), and composite resin buildups (Opallis, FGM; Joinville, SC, Brazil) were placed in increments of 2 mm each. Each increment was light cured for 40 s with an LED light-curing unit set at 1200 mW/cm^2^ (Valo, Ultradent; South Jordan, UT, USA). A single operator performed all bonding procedures.

After 24 h or 2 years, adhesive-dentin bonded sticks (cross-sectional area around 0.9 mm^2^) were prepared using a slow-speed diamond saw (Isomet) and measured by a digital caliper (Digimatic Caliper, Mitutoyo, Tokyo, Japan) to calculate the bond strength in MPa. The number of adhesive-dentin bonded sticks showing pre-test failure (PTF) during specimen preparation was recorded for each tooth.^54,56^ Two adhesive-dentin bonded sticks per tooth from each experimental group were used to evaluate the in-situ degree of conversion within the adhesive/hybrid layers. Three adhesive-dentin bonded sticks per tooth were used to evaluate nanoleakage (24 h and after 2 years), while the remaining adhesive-dentin bonded sticks were tested for microtensile bond strength.

### Microtensile Bond Strength Tests (μTBS)

After 24 h or 2 years of water storage, the adhesive-dentin bonded sticks were fixed to a Geraldeli jig^48^ using cyanoacrylate glue and stressed under tension (Instron; Enfield, CT, USA) at a crosshead speed of 1.0 mm/min until fracture occurred. The μTBS (MPa) was calculated by dividing the applied force (N) at failure by the bonded area. The fracture mode of the adhesive-dentin bonded sticks was examined with a light microscope at 100X magnification (Olympus SZ40; Tokyo, Japan) and categorized as cohesive (C, failure exclusively within the dentin or the resin composite) or adhesive/mixed (A/M, failure at the adhesive-dentin interface or with partial cohesive failure of the neighboring substrates). Specimens with PTF were included in the tooth mean for statistical analysis.

### Nanoleakage (NL)

Three bonded sticks per tooth from each storage period were not used in the μTBS test, but were immersed in an aqueous solution of ammoniacal silver-nitrate solution for 24 h, followed by 8 h in a photo-developing solution under a fluorescent lamp.^60^ The specimens were wet-polished using SiC paper and diamond paste (Buehler). The adhesive-dentin bonded interfaces were observed using a field-emission scanning electron microscope (VEGA 3 TESCAN, Shimadzu; Tokyo, Japan) at 15 kV in the backscatter mode. The amount of silver-nitrate uptake within the adhesive layer and hybrid layer of each adhesive-dentin bonded stick was measured in three regions (5 x 5 μm) of the bonded stick.^54^ The acquisition mode of the images and the percentage of silver-nitrate uptake were calculated according to Hass et al.^28^ ImageJ software (National Institutes of Health; Bethesda, MD, USA) was used to calculate the percentage of silver-nitrate uptake within the hybrid layers in each specimen.

### In-situ Degree of Conversion (DC) by micro-Raman Spectroscopy

Adhesive-dentin bonded sticks (n = 2 per tooth) were prepared as described by Hass et al^28^ and the in-situ degree of conversion of the adhesive interface was measured within the hybrid layer using a dispersive micro-Raman spectrometer/microscope (XploRA ONE Raman microscope, HORIBA Scientific; Piscataway, NJ, USA). Prior to taking measurements, the micro-Raman spectrometer was calibrated to zero. The micro-Raman spectrometer was configured to use with a 638-nm diode laser (1-µm spot diameter) at 100 mW, an X100/0.90 NA air objective, and at 600 lines/mm of grating. The bonded interface was scanned from 400 to 2200 cm^-1^, with a 30-s accumulation time and 6 co-additions. The spectra were acquired at three different sites for each specimen in the middle of the hybrid layer, and the values were averaged for statistical purposes. Post-processing of the spectra was performed using the Opus Spectroscopy Software version 6.5 (Bruker; Billerica, MA, USA). Additionally, spectra of the uncured adhesives were acquired with the method previously described.

The ratio of the double-bond content of monomer:polymer in the uncured and cured adhesive was quantified by calculating the ratio of the aliphatic C=C (vinyl) absorption (1638 cm^-1^) to the aromatic C=C absorption (1608 cm^-1^) signals for both polymerized and unpolymerized samples. DC was calculated using the following formula:

DC (%) = (1–[R_cured_/R_uncured_]) × 100

where “R” is the ratio of aliphatic and aromatic peak intensities at 1638 cm^-1^ and 1608 cm^-1^ in cured and uncured adhesives.^28^ In addition, more intense peaks were observed for all materials, and the corresponding chemical bonding was recorded. The mean values were used for statistical analysis.

### Chemical Interaction Analysis by micro-Raman Spectroscopy

Thirty-two teeth were prepared and restored (n=2), according to the experimental groups. After 24-h water storage, each restored tooth was sectioned into two slices using a slow-speed diamond saw (Isomet). Adhesive-dentin bonded slices were polished with 1500-grit SiC paper for 30 s before being analysed. The chemical profile of adhesive-dentin interfaces was examined with the same dispersive micro-Raman spectrometer/microscope (XploRA ONE Raman microscope) in the same configuration. Spectra were acquired at 3 random sites from the top of the hybrid layer to the underlying dentin at 1-μm intervals.^42^ Eleven spectra were acquired for each site via a computer-controlled x-y-z stage. The acquired spectra were analysed by Labspec 6 software (HORIBA Scientific). The spectra were obtained in triplicate and a comparison was carried out by spectra subtraction for qualitative analysis. The band ratio between the intensity of the pyridinium ring and phenyl vibrations (1032 cm^-1^/1003 cm^-1^) was used to obtain information on cross-linking in the different experimental groups.

### Statistical Analysis

For biofilm assay, data were analyzed using the Kruskal-Wallis test, and the pH of culture media was analyzed using one-way ANOVA. Approximately 19 to 23 adhesive-dentin bonded sticks were obtained per tooth, including the PTFs. The Shapiro-Wilk test was used to assess whether the data from these tests followed a normal distribution. Bartlett’s test was performed to determine the validity of the assumption of equal variances. The mean μTBS (MPa), NL (%), and in-situ DC (%) of all adhesive-dentin bonded sticks from the same tooth were averaged for statistical purposes. Therefore, the experimental unit used in this study was the tooth. The value attributed to PTF specimens was 4.2 MPa, as indicated by previous studies.^28,54^ The μTBS (MPa), NL (%), and in-situ DC (%) means for every test group were calculated from the average of the seven teeth used per group. The μTBS and NL data were analyzed using four-way ANOVA (treatment vs adhesive system vs adhesive strategy vs storage time). The data on in-situ DC and cross-linking rate were analyzed using three-way ANOVA (treatment vs adhesive system vs adhesive strategy). The cross-sectional area was also statistically evaluated using four-way ANOVA. For all adhesive properties, after ANOVA, the data were subjected to post-hoc Tukey’s test with significance set at 0.05.

## Results

### Antibacterial Properties

#### Microbiological susceptibility tests

The MIC of the TCL extract which had an antimicrobial effect against *S. mutans* (UA 159) was 2 mg/ml. Therefore, 5xMIC was equal to 10 mg/ml and 10xMIC was equal to 20 mg/ml (Table 2). The MBC values were similar to those observed for the MIC, indicating bactericidal potential. For chlorhexidine, the MIC was identified as 0.006 mg/ml.

**Table 2:** 96-well plate microdilution method for determining minimum inhibitory concentration

Solution/concentration	mg/ml
Chlorhexidine	0.006
1xMIC	2.0
5xMIC	10.0
10xMIC	20.0

#### Antibiofilm activity of TCL extract against Streptococcus mutans

**Fig 1:**
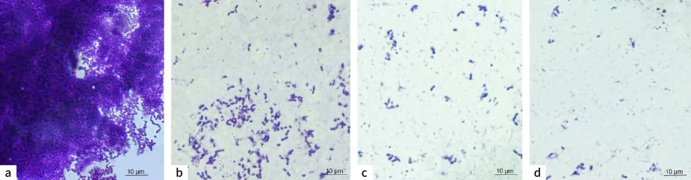
Crystal violet staining of *Streptococcus mutans* cells treated with different concentrations of TCL extract primer. a) Biofilm formation of *S. mutans*. After incubation for 24 h, biofilm inhibition of the *S. mutans* cells was observed at different concentrations of TCL. b) 1xMIC = minimum inhibitory concentration of *Terminalia catappa* L. c) 5xMIC: 5 times the minimum inhibitory concentration. d) 10xMIC: 10 times the minimum inhibitory concentration. Greater inhibition was observed using higher concentrations.

As observed in Fig 1, biofilm production of *S. mutans* was abundant in the control group without TCL extract (Fig 1a). In contrast, the 1xMIC (2 mg/ml), 5xMIC (10 mg/ml) and 10xMIC (20 mg/ml) groups showed significant biofilm inhibition. This was more evident with the higher concentrations (Figs 1c and 1d).

**Fig 2:**
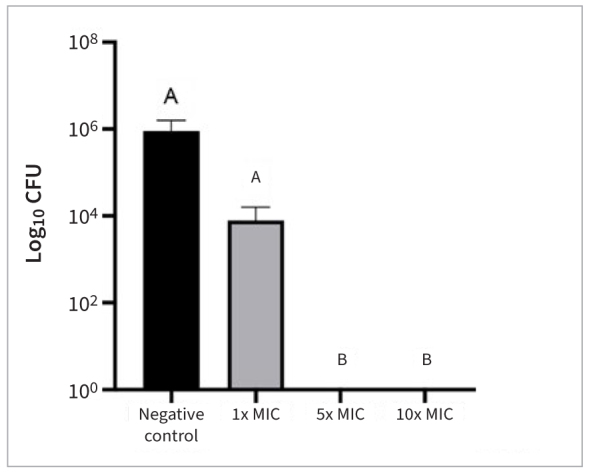
CFU count of 24-h *Streptococcus mutans* biofilms exposed to different concentrations of TCL extract. Different letters indicate statistically significant differences among groups (p < 0.001).

**Fig 3:**
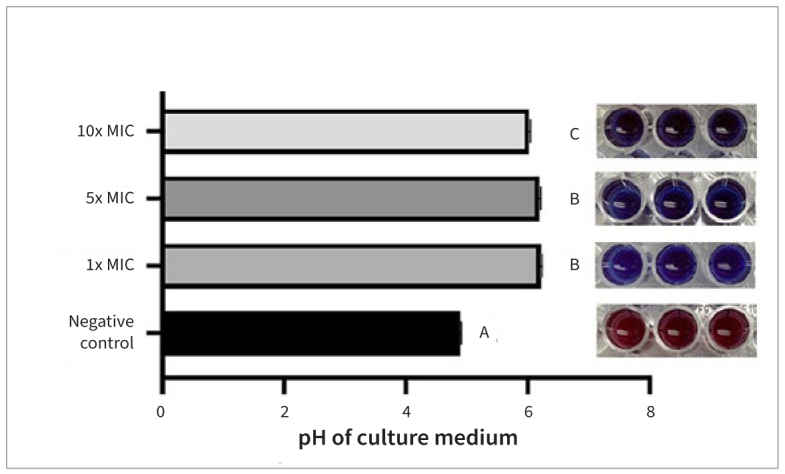
Medium acidification after 24 h of biofilm formation and resazurin assay to illustrate *S. mutans* metabolic activity in the negative control group (not treated), red color. Different letters indicate statistically significant differences in pH of culture medium among groups (p < 0.001).

Considering cell viability, the 5xMIC and 10xMIC groups showed significant inhibition of biofilm formation (p < 0.001), similar to CHX. The findings on metabolic activity and pH of culture media, which indicated biofilm acidogenicity, showed the lowest pH and the greatest metabolization when no treatment was applied to the cariogenic biofilms (Figs 2 and 3).

### Microtensile Bond Strength (μTBS)

The most common failure pattern in all experimental groups was adhesive/mixed (Table 3). The cross-sectional area in the present study was 0.9 ± 0.2 mm^2^ (Table 4). The effect of cross-sectional area on the μTBS among the groups was evaluated as recommended by Armstrong et al.^5^ The results showed no significant difference between groups (p > 0.32).

**Table 3:** Number of specimens (%) according to fracture mode

Adhesive		Control						Extract concentration																	
								1xMIC						5xMIC						10xMIC					
		ER			SE			ER			SE			ER			SE			ER			SE		
		A/M	C	PTF	A/M	C	PTF	A/M	C	PTF	A/M	C	PTF	A/M	C	PTF	A/M	C	PTF	A/M	C	PTF	A/M	C	PTF
SBU	24h	110 (98)	0 (0)	2 (2)	105 (96)	4 (4)	0 (0)	106 (99)	1 (1)	0 (0)	103 (98)	1 (1)	1 (1)	106 (100)	0 (0)	0 (0)	110 (93)	0 (0)	8 (7)	112 (100)	0 (0)	0 (0)	102 (97)	2 (2)	1 (1)
	2y	130 (90)	0 (0)	10 (10)	115 (96)	0 (0)	5 (4)	124 (100)	0 (0)	0 (0)	134 (0)	0 (0)	0 (0)	118 (98)	0 (0)	2 (2)	120 (0)	0 (0)	0 (0)	114 (99)	0 (0)	1 (0)	110 (96)	2 (2)	2 (2)
FBU	24h	108 (95)	0 (0)	5 (5)	102 (97)	2 (2)	1 (1)	101 (98)	2 (2)	0 (0)	100 (96)	0 (0)	3 (4)	107 (97)	2 (2)	1 (1)	116 (97)	0 (0)	3 (3)	106 (99)	1 (1)	0 (0)	116 (94)	0 (0)	5 (6)
	2y	130 (96)	0 (0)	5 (4)	120 (92)	0 (0)	10 (8)	110 (100)	0 (0)	0 (0)	128 (98)	2 (2)	0 (0)	129 (96)	2 (2)	2 (2)	135 (100)	0 (0)	0 (0)	140 (96)	4 (3)	0 (0)	150 (100)	0 (0)	0 (0)

SBU: Scotchbond Universal Adhesive; FBU: Futurabond U adhesive; ER: etch-and-rinse; SE: self-etch; A/M: adhesive/mixed fracture mode; C: cohesive fracture mode; PTF: pre-test failures.

**Table 4:** Means and standard deviations of microtensile bond strength to dentin (MPa) for all experimental groups (*)

Adhesive	Control				Extract concentration											
					1xMIC				5xMIC				10xMIC			
	ER		SE		ER		SE		ER		SE		ER		SE	
	24 h	2 y	24 h	2 y	24 h	2 y	24 h	2 y	24 h	2 y	24 h	2 y	24 h	2 y	24 h	2 y
SBU	44.2 ± 4.3^Ac^	35.1 ± 2.2^Ad^	43.3 ± 3.2^Ac^	34.9 ± 2.8^Ad^	47.1 ± 4.7^Ab^	46.8 ± 2.3^Ab^	47.3 ± 4.8^Ab^	47.5 ± 2.7^Ab^	48.4 ± 5.2^Ab^	47.2 ± 3.6^Ab^	49.1 ± 5.0^Ab^	48.4 ± 3.0^Ab^	52.2 ± 5.1^Aa^	51.9 ± 2.7^Aa^	51.7 ± 4.7^Aa^	50.2 ± 2.6^Aa^
FBU	33.3 ± 4.4^Bc^	23.9 ± 3.0^Bd^	31.6 ± 4.4^Bc^	24.0 ± 3.0^Bd^	35.8 ± 5.4^Bb^	33.9 ± 3.5^Bb^	36.6 ± 5.1^Bb^	36.7 ± 3.5^Bb^	40.6 ± 4.3^Bb^	38.2 ± 3.6^Bb^	41.8 ± 3.2^Bb^	40.0 ± 3.2^Bb^	46.1 ± 3.6^Ba^	45.44 ± 2.5^Ba^	44.2 ± 3.9^Ba^	44.8 ± 2.0^Ba^

SBU, Scotchbond Universal Adhesive; FBU, Futurabond U adhesive; ER, etch-and-rinse; SE, self-etch. (*) Different superscript capital letters indicate statistically significant differences between adhesives in each column. Different superscript lowercase letters indicate statistically significant differences between the groups for each adhesive (four-way repeated measures ANOVA; Tukey’s test, p < 0.05).

The four cross-product interactions as well as the triple cross-product interactions did not yield significant differences for μTBS values (p > 0.11). However, a significant difference was observed in the two double cross-product interactions (treatment vs adhesive and treatment vs storage time) (Table 4; p < 0.0001). At 24 h, the application of the TCL extract at all concentrations significantly increased the μTBS for both adhesives when compared with the control groups (Table 4; p < 0.0001). Notably, the statisitcally significantly highest μTBS were found with the highest multiple of MIC (10xMIC) vs the lower multiples of MIC (1xMIC and 5xMIC; Table 4).

After 2 years of water storage, the µTBS of the control groups significantly decreased compared to after 24 h of storage. On the other hand, after2 years, TCL extract at all concentrations maintained the μTBS for both adhesives when compared to the results after 24 h (Table 4; p < 0.0001). Once again, the statistically significantly highest μTBS at both evaluation times were found with the highest concentration of TCL (10xMIC), in comparison with the two lower concentrations (1xMIC and 5xMIC; Table 4). In all comparisons, SBU showed higher μTBS than did FBU, regardless of the adhesive strategy (Table 4; p = 0.0001).

### Nanoleakage (NL)

**Fig 4:**
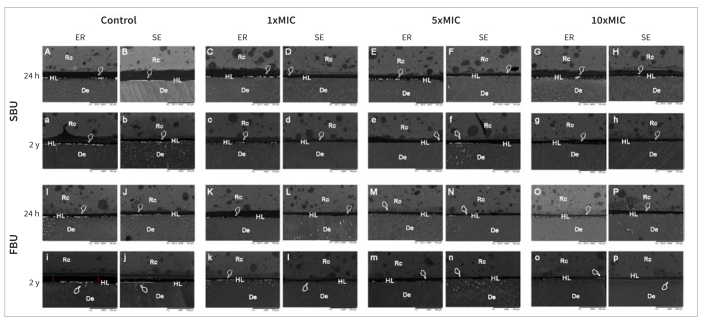
Backscattered scanning electron micrographs of the adhesive interface of all experimental groups immediately (24 h) or after 2 years of water storage. Silver-nitrate infiltration was observed for all experimental groups (white pointer). For both adhesives, less silver-nitrate infiltration was found after 24 h (capital letters) than after 2 years of water storage (lowercase letters). However, these deposits were more evident in the control groups, with the presence of debonded areas (red line in image “i”). When *Terminalia catappa* L. extract was used, independent of the concentration, the silver-nitrate concentration was less evident after 24 h or 2 years of water storage when compared to control groups. SBU = Scotchbond Universal adhesive; FBU = Futurabond U; ER = etch-and-rinse; SE = self-etch; 1xMIC: minimum inhibitory concentration of *Terminalia catappa* L.; 5xMIC: 5 times the minimum inhibitory concentration; 10xMIC: 10 times the minimum inhibitory concentration (Rc = resin composite; HL = hybrid layer; De = dentin).

The nanoleakage data are presented in Fig 4 and Table 5. No significant difference was found for the four cross-product interactions, the triple cross-product interactions (p > 0.36), and the four double cross-product interactions (p > 0.32). However, a significant difference was observed in the two double cross-product interactions (treatment vs adhesive and treatment vs storage time) (Table 5; p < 0.0001).

**Table 5:** Means and standard deviations of nanoleakage (%) for all experimental groups (*)

Adhesive	Control				Extract concentration											
					1xMIC				5xMIC				10xMIC			
	ER		SE		ER		SE		ER		SE		ER		SE	
	24 h	2 y	24 h	2 y	24 h	2 y	24 h	2 y	24 h	2 y	24 h	2 y	24 h	2 y	24 h	2 y
SBU	14.3 ± 1.8^Ad^	21.0 ± 2.7^Ae^	14.2 ± 2.1^Ad^	21.6 ± 2.0^Ae^	9.5 ± 3.2^Ab^	13.5 ± 2.5 ^Acd^	9.8 ± 3.2^Ab^	13.7 ± 1.4^Acd^	9.4 ± 2.5^b^	13.1 ± 1.8^Acd^	9.7 ± 2.7^Ab^	13.3 ± 2.0^Acd^	7.1 ± 2.5^Aa^	10.4 ± 2.2^Ab^	7.1 ± 2.5^Aa^	10.2 ± 2.0^Ab^
FBU	14.8 ± 2.1^Ad^	22.1 ± 2.4^Ae^	14.7 ± 2.5^Ad^	22.0 ± 2.1^Ae^	9.1 ± 2.9^Ab^	13.1 ± 1.9 ^Acd^	9.2 ± 2.9^Ab^	13.6 ± 2.3^Acd^	9.9 ± 3.2^b^	13.0 ± 1.6^Acd^	9.8 ± 2.7^Ab^	12.8 ± 2.1^Acd^	7.2 ± 2.3^Aa^	10.9 ± 2.4^Abc^	7.2 ± 2.5^Aa^	10.8 ± 2.1^Ab^

SBU, Scotchbond Universal Adhesive; FBU, Futurabond U adhesive; ER, etch-and-rinse; SE, self-etch. (*) Different superscript capital letters indicate statistically significant differences between adhesives in each column. Different superscript lowercase letters indicate statistically significant differences between the groups for each adhesive (four-way repeated measures ANOVA; Tukey’s test, p < 0.05).

After 24 h, TCL extract at all concentrations significantly decreased the NL for both adhesives when compared with the control groups (Table 5; p = 0.0001). However, after 2 years of water storage, all groups showed a significant increase in NL compared to the control groups (Table 5; p = 0.00001). Notably, the control groups exhibited significantly higher NL for both adhesives when compared to the application of TCL extract at all concentrations after 2 years of water storage (Table 5; p = 0.00001). It is worth mentioning that significantly lower NL values at both evaluation times were found when the highest TCL concentration (10xMIC) was used vs lower concentrations (1xMIC and 5xMIC; Table 5). In all comparisons, both adhesives showed similar NL values (Table 5).

### In-situ Degree of Conversion (DC)

No significant difference was found for the triple cross-product interaction, nor for the two double cross-product interactions (treatment vs adhesive strategy and adhesive vs adhesive strategy; p > 0.17). However, a significant difference was observed in the double cross-product interaction (treatment vs adhesive) (Table 6; p < 0.02), as well as the main factors of treatment and adhesive (p = 0.00002 and p = 0.000001, respectively). Additionally, the main factor adhesive strategy was not statistically significantly different (p = 0.63). TCL extract at all concentrations significantly increased the DC values for both adhesives when compared with the control groups (Table 6; p = 0.00002). In all comparisons, SBU showed higher DC values than FBU, regardless of the adhesive strategy (Table 6; p = 0.000001).

**Table 6:** Means and standard deviations of in-situ degree of conversion (%) for all experimental groups (*)

Adhesive	Control		Extract concentration					
			1xMIC		5xMIC		10xMIC	
	ER	SE	ER	SE	ER	SE	ER	SE
SBU	62.7 ± 2.8^Ab^	62.1 ± 2.0^Ab^	67.0 ± 2.8^Aa^	66.3 ± 1.2^Aa^	66.0 ± 2.6^Aa^	67.7 ± 1.9^Aa^	66.2 ± 2.4^Aa^	66.1 ± 2.6^Aa^
FBU	57.0 ± 1.3^Bb^	57.4 ± 2.3^Bb^	61.9 ± 2.7^Ba^	61.0 ± 2.1^Ba^	61.2 ± 1.7^Ba^	63.6 ± 2.9^Ba^	61.0 ± 2.3^Ba^	61.5 ± 2.7^Ba^

SBU, Scotchbond Universal; FBU, Futurabond U. (*) Different superscript capital letters indicate statistically significant differences between adhesives in each column. Different superscript lowercase letters indicate statistically significant differences between the groups for each adhesive (three-way repeated measures ANOVA; Tukey test, p < 0.05).

### Chemical Interaction Analysis by micro-Raman Spectroscopy

**Fig 5:**
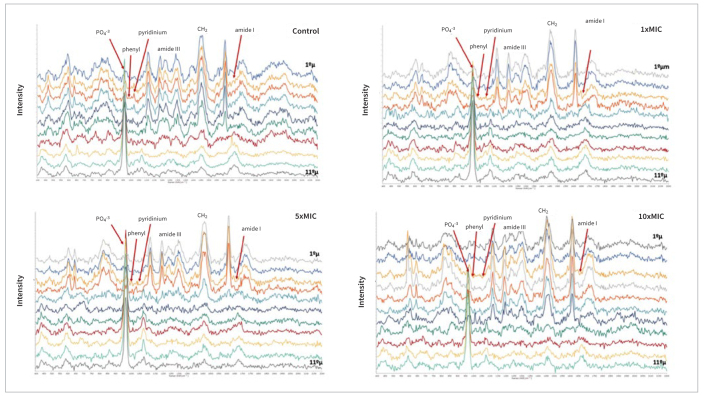
Chemical profile with the representative micro-Raman spectra acquired across the adhesive-dentin interface created by the adhesives tested with the application of the different concentrations of *Terminalia catappa* L. Spectra starting from the middle of the hybrid layer (top spectra) to the underlaying dentin (bottom spectra) show the region of interest covering relative peaks assigned to methacrylate monomers, mineralized dentin, and demineralized dentinal collagen in the hybrid layer. The indicated peaks – pyridinium ring at 1031 cm^-1^ and phenyl vibrations at 1001 cm^-1^ (1C; 1031 cm^-1^ / 1001 cm^-1^) – were used to measure the relative cross-linking effect of *Terminalia catappa* L. on collagen (Table 7).

Figure 5 shows representative micro-Raman spectra of the adhesive-dentin interface created by SBU in etch-and-rinse mode in the control group and in the TCL extract groups. The recorded spectra covered the range of 400 to 2200 cm^-1^, which includes the fingerprint region associated with the hybrid layer and dentin collagen bands. Peaks corresponding to the adhesives were identified (C—O—C at 1113 cm^-1^, C ═ C at 1610 cm^-1^, and C∙O at 1720 cm^-1^). The relative decrease in the intensity of these peaks indicated the transition through dentin.

Dentin collagen bands were also evident, with peaks at amide I (1667 cm^-1^), amide III (1243 cm^-1^ and 1273 cm^-1^), the phenyl group (1003 cm^-1^) from the aromatic ring of phenylalanine residues in collagen, and the pyridinium ring (1032 cm^-1^), which represents a trivalent amino acid cross-linking residue. Additionally, functional groups of carbonated apatite (phosphate at 962 cm^-1^ and carbonate at 1072 cm^-1^) were detected within the mineralized dentin (Fig 5). Only one example of micro-Raman spectra of the adhesive-dentin interface from one experimental group was included, considering the observed similarities among the spectra in the former and other experimental groups.

No significant difference was found for triple or double cross-product interactions (p > 0.12), nor for the main factors adhesive and adhesive strategies (p = 0.52 and p = 0.78, respectively). However, a significant difference was observed in the main factor treatment (Table 7; p = 0.00001). For all groups treated with TCL, an increase in the cross-linking rate was observed, as seen in the band ratio of pyridinium (1031 cm^-1^) / phenyl (1001 cm^-1^) values for both adhesives when compared with the control groups (Table 7; p = 0.00001).

**Table 7:** Mean and standard deviation of crosslinking rate from pyridinium (1031 cm^-1^)/phenyl (1001 cm-1) for the different experimental groups (*)

Adhesive	Control		Extract concentration					
			1xMIC		5xMIC		10xMIC	
	ER	SE	ER	SE	ER	SE	ER	SE
SBU	1.21 ± 0.09^Ac^	1.11 ± 0.08^Ac^	1.47 ± 0.09^Ab^	1.43 ± 0.09^Ab^	1.69 ± 0.09^Aab^	1.68 ± 0.12^Aab^	1.88 ± 0.12^Aa^	1.92 ± 0.10^Aa^
FBU	1.23 ± 0.09^Ac^	1.14 ± 0.07^Ac^	1.49 ± 0.11^Ab^	1.44 ± 0.12^Ab^	1.71 ± 0.08^Aab^	1.67 ± 0.14^Aab^	1.90 ± 0.13^Aa^	1.86 ± 0.09^Aa^

SBU, Scotchbond Universal Adhesive; FBU, Futurabond U adhesive. (*) Different superscript capital letters indicate statistically significant differences between adhesives in each column. Different superscript lowercase letters indicate statistically significant differences between the groups for each adhesive (three-way repeated measures ANOVA; Tukey’s test, p < 0.05).

However, the significantly highest cross-linking rate values were found with the highest MIC concentration (10xMIC) compared to the 1xMIC group. The 5xMIC group showed intermediary cross-linking rate values (Table 7). Additionally, for all groups treated with TCL, a slight shift of amide III to a lower wavenumber was observed. For the 1xMIC and 5xMIC groups, it was possible to observe a slight shift of amide III from ~1243 cm^-1^ to a lower wavenumber of ~1238 cm^-1^. For 10xMIC, a slight shift of amide III from ~1243 cm^-1^ to a lower wavenumber of ~1225 cm^-1^ was observed.

## Discussion

Currently, plant extracts are a promising option for preventing and treating infections.^55,61,64^ Previous studies have shown that polar fractions obtained from TCL leaves are effective against bacteria and fungi.^14,57,61^ The present pioneering study investigated the effect of TCL extract on *S. mutans* by incorporating the different concentrations of it into the restorative procedure.

The microbiological efficacy (MIC and MBC) was demonstrated at a TCL extract concentration of 2 mg/ml, leading to a 99.9% reduction in the initial bacterial population. This antibacterial effect was also confirmed in antibiofilm tests (Figs 1 to 3), particularly at higher concentrations, supporting our initial hypothesis. Incorporating the extract into the primer could be an option to prevent biofilm formation at the restoration interface and reduce the risk of secondary caries.

Using chemical analyses, previous studies have confirmed the presence of hydrolyzable tannins (ellagic acid, punicalin and punicalagin), gallic acid, and flavonoid C-glycosides in TCL extract.^61^ Hydrolyzable tannins are the primary chemical constituents of TCL leaves, and they are associated with antitumor and antioxidant activity, along with strong antimicrobial activity against bacteria and yeast.^11,35,38^ The antimicrobial action is attributed to hydrolyzable tannin’s involvement in protein precipitation and the removal of metal and hydrogen ions from microbial enzymes, thereby altering crucial metabolic processes in the microorganisms.^24,46,61^ Their antimicrobial activity is reflected in the results of the present study.

However, despite this antimicrobial effect, it is important to evaluate the effect of using different concentrations of TCL extract in the adhesive procedure, mainly because the adhesive-dentin interface can be jeopardized according to the type and concentration of the botanical agent.^20,21,25,40^

In the present study, regardless of the concentration used (1xMIC, 5xMIC, or 10xMIC), the TCL extract significantly increased the immediate bond strength and effectively preserved it, while simultaneously preventing NL after 2 years for both adhesives when compared to the control groups. Thus, the second hypothesis was accepted. As previously described, the TCL extract contains natural and biocompatible hydrolyzable tannins and flavonoid C-glycosides^10,67^ with excellent biomodification potential.^3,7,68^ The fractions punicalagin and ellagic acid are particularly relevant, due to their ability to interact with dentin collagen.^61^ The free galloyl group present in these fractions has been previously shown to play a significant role in cross-linking and protecting collagen from degradation.^29^ This group is recognized for its crucial function in promoting strong interactions between collagen and polyphenols.^59^ Specifically, the galloyl group provides numerous collagen-reactive groups, including aromatic and phenolic hydroxyl groups.^36^ The aromatic groups enhance hydrophobic interactions through π-π interactions with the hydrophobic region of aromatic groups in polyphenols, as found in the TCL extract, and the hydrophobic side chains of amino acids in collagen.^29,36^ Additionally, the inclusion of phenolic hydroxyl groups increases the potential for hydrogen bond formation with carbonyl and free amino groups in collagen.^30^ Previously, a shift in the wavenumber of amide bands has been demonstrated and attributed to hydrogen bonds mediated by polyphenols on dentin collagen using FTIR.^30^ These shifts likely account for the slight changes in amide III bands observed in the chemical profile by micro-Raman spectroscopy.

Interactions with collagen provided by other functional groups in TCL extract, or even through other chemical mechanisms, are not precluded. For instance, interactions through covalent bonds would depend on the autoxidation of phenolic hydroxyl groups into orthoquinones to react with amino and carbonyl on collagen through a Schiff-base reaction.^30,66^ However, due to the limited mobility of most functional groups in all TCL extract fractions, these interactions are less likely to occur.^29,59^

In the present study, in an attempt to find more evidence of the role of TCL extract in significantly increasing the adhesive properties of dentin, the cross-linking rate induced in dentin collagen by the TCL extract was also quantified. The band ratio of pyridinium (~1031 cm^-1^), which represents a trivalent amino acid cross-linking residue, and that of the phenyl group (~1001 cm^-1^) from the aromatic ring of phenylalanine residues in collagen were used.^17^ A significant difference was observed for all groups in which TCL extract was applied compared to the control group (distilled water), confirming the interactions mediated by TCL extract with collagen, as described above. This leads to acceptance of the third hypothesis. Thus, we hypothesized that this significant increase in the adhesive properties of dentin^44^ could be explained by the cross-linking effect of TCL extract when applied to dentin. More important than an immediate collagen cross-linking effect is the maintenance of this effect over time. It is noteworthy that, regardless of concentration, the application of TCL extract used as a primer on dentin preserved the adhesive properties even after 2 years of water storage when compared with the control group. However, future studies need to assess the long-term impact of the TCL extract primer on dentin collagen cross-linking.

Additionally, after both storage periods, a positive correlation was observed between TCL concentration and μTBS, with the significantly highest values observed with the highest concentration (10xMIC). This correlation agrees with previous studies, suggesting a dependency on the number of reactive phenolic groups and the availability of reactive groups in each molecule which can interact with hydroxyl, carboxyl, amine, or amide groups in dentin collagen fibrils.^1,26,27^ This correlation is supported by the chemical profile evaluation of the adhesive interface, indicating a stronger cross-linking interaction between dentin and the TCL extract when a higher concentration was used. Therefore, it was possible to confirm that with the highest concentration of TCL extract in the 10xMIC group, more molecules were available for bonding with collagen. Consequently, a higher number of cross-links formed, resulted in higher μTBS.

A noteworthy characteristic of the TCL extract is its dark color, as previously described for other natural plant extracts.^18,44^ However, the final solutions of 1xMIC, 5xMIC, and 10xMIC remain transparent. Additionally, all specimens treated with the extract at different concentrations did not exhibit any color alteration during the restorative procedure, which is advantageous for clinical application. The oxidative properties and high-molecular-weight polyphenols of several flavonoids of natural origin, such as proanthocyanidins, can induce several color alterations in dentinal substrates.^44^ Furthermore, at higher concentrations (≥5%), the polyphenols can potentially interfere with the polymerization reaction of adhesives.^25,40^ However, this interference was not observed in the present study.

After 24 h, independent of the concentration, the TCL extract unexpectedly resulted in decreased NL for both adhesives and adhesive strategies. The protocols tested in this study cannot alter the hydrophilic nature of the adhesive, improve water/solvent evaporation, or produce less permeable adhesive interfaces. Therefore, it was not expected that the application of the TCL extract would lead to an adhesive interface with reduced NL. However, this result was observed in all experimental groups compared with the control group.

Although not evaluated in the present study, a plausible explanation for the above findings may be related to the ability of flavonoids to modify the moisture level and permeability of dentin.^37^ This effect may be attributed to the high density of crosslinks formed by flavonoids in dentin, which reduces the surface hydrophilicity of dentin,^32^ and the intrinsic hydrodynamics of dentinal tubules.^1^ Furthermore, flavonoids in synergy with the solvent used in this study may have improved the displacement of proteoglycans within the collagen network,^19,37^ which facilitated the infiltration of resin monomers on the dentin surface.

Although TCL extract improved the µTBS to dentin in this study, some signs of NL were present in all groups. It cannot be expected that the application of flavonoids would produce a NL-free adhesive-dentin interface. This is because the flavonoid agents do not alter the inherently hydrophilic nature of the adhesives, improve water/solvent evaporation, or reduce the permeability of adhesive interfaces.^31^

Although it has been demonstrated that flavonoids could prevent the degradation of organic components of the hybrid layer,^19^ as observed in the present study, lower NL results were observed after 2 years of water storage when different concentrations of TCL extract were used in comparison with control groups. However, it is important to note that they cannot prevent polymer degradation over time. With time, water sorption and polymer swelling facilitate leaching of hydrophilic monomers within the adhesive blends,^8,39^ thus contributing to an increase in the number of voids within the polymer network^8,54^ and exposing additional collagen that is vulnerable to attack by proteolytic enzymes.^47^

Regarding the DC, the results of the present study showed that, regardless of the concentration, a significant increase in DC was observed with TCL extract compared to the control group. Additionally, considering the changes in dentin moisture due to the high-density crosslinking of flavonoids within collagen and the therapeutic effect on dentin of crosslinking molecules, some authors have suggested a positive copolymerization of flavonoids with the bonding agent, resulting in ester-type chemical bonds that improve the mechanical properties of the adhesive layer.^13,41,70^ Therefore, these factors may have increased the monomer conversion of the adhesive, independent of the concentration of the TCL extract, explaining the higher DC values observed in the present study.

Two universal adhesives were evaluated in the present study. For all comparisons, SBU outperformed FBU, regardless of the adhesive strategy. This difference in performance may be explained by the absence of the functional monomer 10-MDP in FBU,^65^ while SBU contains 10-MDP and a methacrylate-modified polyalkenoic acid copolymer monomer,^50,69^ enhancing its chemical interaction with the tooth structure.^2^ Therefore, this could be considered another factor that positively influences the performance of this adhesive.

Some limitations need to be addressed. Regarding the antimicrobial properties, although we chose the main microorganism (*S. mutans*) associated with caries to evaluate how the plant extract behaves in terms of reduction and/or control the biofilm formation,^15^ the use of only one microorganism is limited and future studies should be conducted to evaluate the antimicrobial potential of TCL extract in preventing secondary caries. Despite the positive effects observed after two years of water storage using TCL extract, as only two universal adhesives were tested in the present study, the observed effect may be adhesive specific. Therefore, future studies need to be conducted to evaluate the effect of TCL extract when used with other universal adhesives. Finally, due to the limitations of any in-vitro study, future clinical trials are needed to better understand the benefits of the use of the TCL extract associated with the adhesive protocol in the inhibition of secondary caries.

## Conclusion

The use of *Terminalia catappa* L. extract showed microbiological efficacy and antibiofilm activity against *Streptococcus* *mutans*, as well as higher cross-linking rates with dentin, while maintaining the long-term stability of the adhesive-dentin interface.

## Clinical relevance:

The use of adhesives containing TCL prevents bonding degradation to dentin and may represent a promising strategy to prevent secondary caries in dentin.
